# Stabilisation of Exotic Tribromide (Br_3_^−^) Anions via Supramolecular Interaction with a Tosylated Macrocyclic Pyridinophane. A Serendipitous Case

**DOI:** 10.3390/molecules25143155

**Published:** 2020-07-10

**Authors:** Álvaro Martínez-Camarena, Matteo Savastano, Carla Bazzicalupi, Antonio Bianchi, Enrique García-España

**Affiliations:** 1 ICMol, Department of Inorganic Chemistry, University of Valencia, C/Catedrático José Beltrán 2, 46980 Paterna, Spain.; alvaro.martinez@uv.es; 2 Department of Chemistry “Ugo Schiff”, University of Florence, Via della Lastruccia, 3-13, 50019 Sesto Fiorentino, Italy; matteo.savastano@unifi.it (M.S.); carla.bazzicalupi@unifi.it (C.B.)

**Keywords:** N-heterocycles, anion complexes, anion-π interactions, crystal structure, Hirshfeld surface analysis

## Abstract

Tetraaza-macrocyclic pyridinophane L-Ts, decorated with a *p*-toluenesulfonyl (tosyl; Ts) group, appear to be a useful tool to provide evidence on how the interplay of various supramolecular forces can help stabilise exotic anionic species such as tribromide (Br_3_^−^) anions. Indeed, crystals of (H_2_L-Ts)(Br_3_)_1.5_(NO_3_)_0.5_ unexpectedly grew from an acidic (HNO_3_) aqueous solution of L-Ts in the presence of Br^−^ anions. The crystal structure of this compound was determined by single crystal XRD analysis. Hydrogen bonds, salt-bridges, anion-π, π-π stacking, and van der Waals interactions contribute to stabilising the crystal lattice. The observation of two independent Br_3_^−^ anions stuck over the π-electron densities of pyridine and tosyl ligand groups, one of them being sandwiched between two pyridine rings, corroborates the significance of anion-π interactions for N-containing heterocycles. We show herein the possibility of detecting anion-π contacts from fingerprint plots generated by Hirshfeld surface analysis, demonstrating the effective usage of this structural investigation technique to further dissect individual contributions of stabilising supramolecular forces.

## 1. Introduction

Since the early years of coordination chemistry, N-heterocyclic ligands have aroused a great deal of interest for their ability to form metal complexes with relevant properties [1]. Moreover, the basic character of heterocyclic nitrogen atoms, which can undergo protonation to form cationic species, and the polarisation of their aromatic π-electrons clouds, have more recently promoted the use of these ligands for the preparation of anion complexes [2,3], as well as for the construction of assemblies of neutral molecules [4].

**Figure 1:**
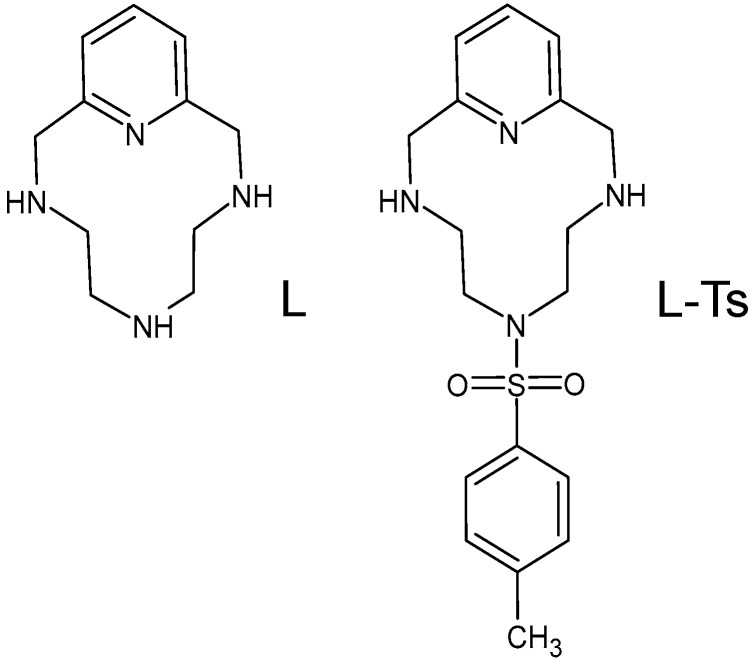
Ligand L and its tosylated derivative L-Ts.

Pyridinophane 3,6,9,15-tetraazabicyclo[9.3.1]pentadeca-1(15),11,13-triene (L, Figure 1) is a member of this family that was first synthesised in 1981 [5]. It was later in the spotlight for its ability to form metal complexes [6,7,8], but its wider success was achieved thanks to its utilisation as a scaffold for the preparation of new materials that have proven effective in a variety of applications. Indeed, L derivatives decorated with different hanging functionalities have been studied for several biomedical (MRI contrast agents [9,10,11,12], antiproliferative treatments [13,14,15,16], radiotherapy [17,18], enzyme mimicking [19,20] and antioxidant activity [21,22,23]), chemosensing [24,25,26,27] and catalytic [28,29,30,31,32,33] applications. Very recently, it has been shown that L and its N-trimethylated derivative are capable of stabilising iodine-dense three-dimensional networks that might be relevant for crystal engineering purposes and the design of solid-state conductors [34].

**Figure 2:**
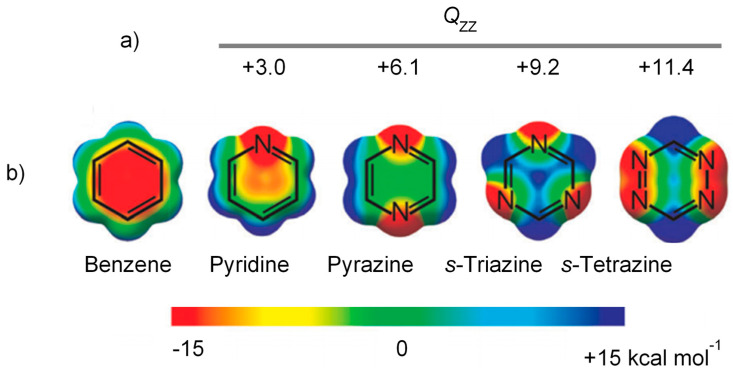
**(a**) *Q*_zz_ quadrupole moments (in Buckinghams) for azines, relative to benzene. (**b**) Total ESPs of benzene and azines, mapped onto the corresponding electron density isosurfaces. Partially reproduced from ref. [35] with permission from the Royal Society of Chemistry.

We have now fortuitously discovered that the mono-tosylated derivative of L (L-Ts, Figure 1) is able to stabilise the exotic tribromide (Br_3_^−^) anion in the solid state by deploying a large variety of supramolecular forces, including controversial and counterintuitive anion-π interactions. It is known that azines may act as π-acid ligands, which can bind anions above their centres according to their positive electrostatic potentials (ESPs) and their molecular quadrupole moments (Figure 2) [35]. For example, we have recently shown that *s*-tetrazine-based ligands can form anion complexes [36] with both inorganic [37,38,39,40,41,42] and organic [43] anions featuring clear anion-π interactions. Spontaneous oxidation of iodide in solution and stabilisation of triiodide (I_3_^−^) anions in the solid state were observed in the presence of these *s*-tetrazine based ligands [38], while bromide was found to interact with the rings of these π-acids, but no oxidation to Br_3_^−^ was detected [39]. Anion-π interactions were also found to play an important role in the formation of highly organised polyiodide complexes [44,45] and iodide based permutable organised frameworks (POF) [46] with a pyridinium-based ligand such as Stoddart’s blue box (BB, cyclobis(paraquat-*p*-phenylene)).

In this paper, we report the crystal structure of (H_2_L-Ts)(Br_3_)_1.5_(NO_3_)_0.5_ (**1**), which appeared unexpectedly from a sample of L containing L-Ts as an impurity. The structure was surprising and very appealing, as it shows that tribromide anions can interact via anion-π interactions not only with the pyridine ring, which is the weakest π-acid among azines, even with benzene rings.

Given our attention to the topic of anion-π interactions and the interest for novel methodologies that may help us disentangle the individual contribution of different supramolecular forces within a crystal packing, like Hirshfeld surface analysis, we ended up examining a certain number of fingerprint plots of anion complexes [44,45,46]. As we discuss the peculiarities of the (H_2_L-Ts)(Br_3_)_1.5_(NO_3_)_0.5_ crystal structure under this viewpoint, we cannot help but notice that typical fingerprint plot features exist whenever an anion-π interaction is formed. Therefore, we will point out such general properties and show how anion-π interactions can be identified at once from both ligand and anion fingerprint plots. This is an important contribution to the appreciation of this kind of interaction for N-heterocyclic ligands meant to assist researchers, and perhaps software in the future, to immediately recognise these contacts from fingerprint plots, exactly as can be done for H-bonds or π-π stacking interactions. 

## 2. Results and Discussion

### 2.1. Crystallographic Results

Crystals of (H_2_L-Ts)(Br_3_)_1.5_(NO_3_)_0.5_ (**1**) were a pleasant and instructive surprise. They were obtained unintentionally when we tried to prepare crystals of a nitrate salt of H_2_L^2+^ by slow evaporation of an aqueous solution of L⋅3HBr in diluted HNO_3_. We were aware that the L⋅3HBr sample contained a small impurity (about 2%) of the mono-tosylated ligand that remained after a not completely exhaustive detosylation of L-Ts_3_ with HBr/CH_3_COOHC, but we did not expect the impurity to be the only solid compound to separate (as **1**) from the concentrated mother liquor. Most likely, the tosyl residue contributed to lowering the ligand solubility, favouring its crystallisation. The formation of Br_3_^−^ was appreciated by a change of colour of the mother liquor that turned from colourless to pale orange after several days of evaporation when the solution had reduced to a small volume. Reasonably, Br_3_^−^ was generated by Br^-^ oxidation with HNO_3_ when the solution became concentrated enough. 

**Figure 3:**
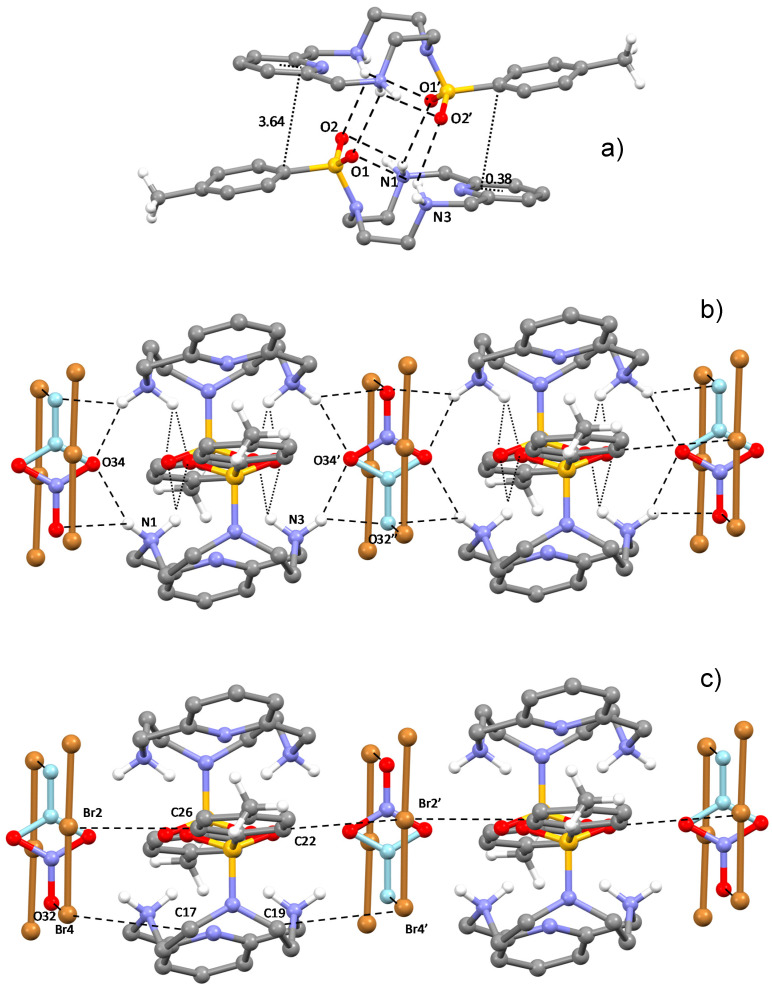
Views of the crystal structure of (H_2_L-Ts)(Br_3_)_1.5_(NO_3_)_0.5_. (**a**) (H_2_L-Ts)^2+^ ligand pairs; distances in Å. (**b**) Interactions formed by NO_3_^−^ anions; only one of the possible motifs compatible with this pseudosymmetric disorder is shown. (**c**) Interactions formed by one of the non-equivalent tribromide anions.

Crystals of **1** are composed of (H_2_L-Ts)^2+^ ligand molecules, protonated on both aliphatic nitrogen atoms and interacting Br_3_^−^ and NO_3_^-^ anions. The diprotonated ligand molecules are associated in head-to-tail centrosymmetric pairs held tightly joined by a network of hydrogen bonds and π-stacking interactions (Figure 3a). Each tosyl group forms a couple of intermolecular hydrogen bonds with the two ammonium groups of the facing (H_2_L-Ts)^2+^ cation and a couple of intramolecular hydrogen bonds with the ammonium groups of its own protonated molecule (Figure 3a, Table 1). These H-bonds are of the bifurcated type, each nitrogen involving the same hydrogen atom in two contacts, giving an overall diamond shape. Pyridine and benzene rings of facing molecules give rise to the π-stacking interactions: the plane of the two rings form an angle of 15.5°, and the benzene atom closest to the pyridine ring plane is the C atom linked to sulphur (3.64 Å), which is located almost above the pyridine centroid (offset 0.38 Å). 

Nitrate participates in the overall packing, supporting intra- and inter-dimer connections. As shown in Figure 3b, NO_3_^−^ stabilises ligand pairs via short hydrogen bonds (Table 1), while, at the same time, actively bridges different pairs into hydrogen bonded ribbons developing along the *a* cell direction. The nitrate anion is disordered, spread over two sets of centrosymmetric atomic sites. Accordingly, only one of the possible motifs compatible with this pseudosymmetry is shown in Figure 3b, but many others may be possible.

The two crystallographically non-equivalent Br_3_^−^ anions have a different structural role. They fulfil a plethora of different interactions preferences of what we could describe as “secondary” anion binding sites of the ligand.

**Figure 4:**
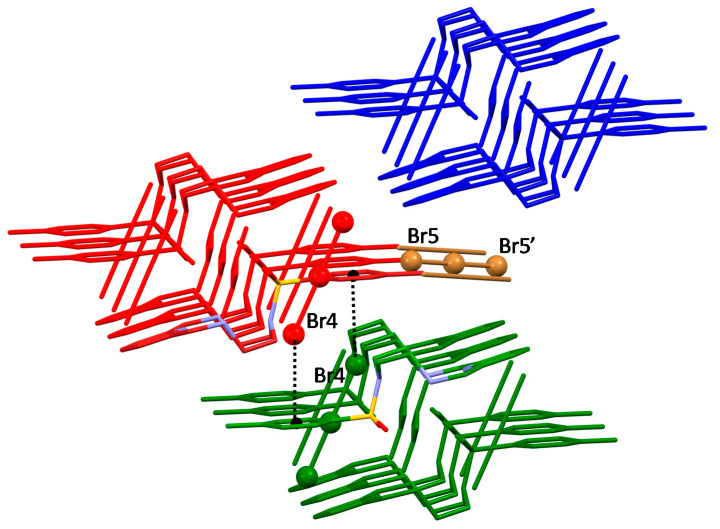
View of the crystal structure of (H_2_L-Ts)(Br_3_)_1.5_(NO_3_)_0.5_ highlighting the end-on anion-π interactions formed by one of the crystallographically non-equivalent Br_3_^−^ anions with benzene rings.

Couples of the first non-equivalent tribromide anions are interposed between ligand pairs along the above-mentioned ribbons, contributing to their stabilisation via electrostatic and CH⋅⋅⋅Br interactions with the protonated ligand molecules (Figure 3c). The latter are the main ligand to anion contacts within these ribbons, which involve benzylic methylenic groups, methylenic groups next to the tertiary amine and ortho aromatic protons of the tosyl group (Table 1). In addition, there is an interesting contact linking a nitrate oxygen and a tribromide anion for each pair (Br4⋅⋅⋅O32 (x + 1,+ y + 1,+ z) 3.05(2) Å, Figure 3c, Table 1). This contact is quite short and unusual, involving two clearly not H-bonded negatively charged species. A possible explanation of this contact could be related to the anisotropy of the electrostatic potential around both Br_3_^−^ and nitrate. It is known that while nucleophilic species can approach the halogen atom along the extension of the covalent bond, electrophiles prefer to approach it along a direction orthogonal to the bond. Nitrate is not easily defined as an electrophilic species, nevertheless, the N-O distance of the oxygen atom involved in this contact is much longer than for the other two oxygen atoms (O32-N33 1.31(2) vs. O34-N33 1.25(2) Å), which indicates a higher sigma character. Accordingly, this region of the NO_3_^-^ anion is most likely depleted of electron density, with an overall positive electrostatic potential that may explain the short Br⋅⋅⋅O contact. These tribromide anions, having an unsymmetrical structure (Br4-Br2 2.64, Br2-Br3 2.48 Å), give rise to end-on anion-π interactions with benzene rings of neighbouring ribbons (Figure 4), so that symmetry-related ribbons are doubly connected by these centred (Br4⋅⋅⋅ring centroid 3.71 Å, offset 0.30 Å) η^6^-type interactions (C⋅⋅⋅Br4 3.88–4.09 Å). 

**Figure 5:**
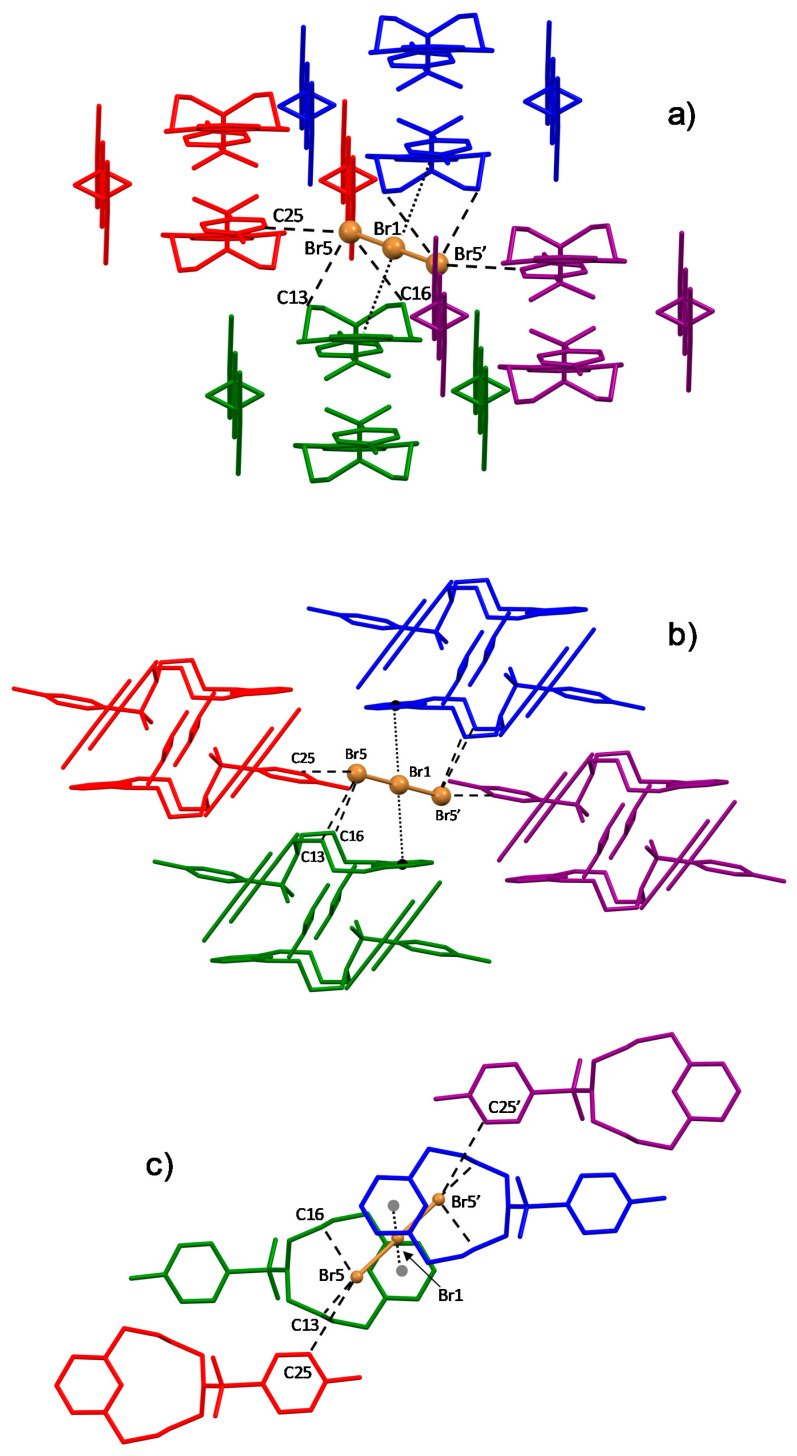
Lateral (**a**) and (**b**) and top (**c**) views of the Br_3_^−^ anion sandwiched between pyridine ligand groups in the crystal structure of (H_2_L-Ts)(Br_3_)_1.5_(NO_3_)_0.5_.

The second non-equivalent tribromide has instead the role of sticking together adjacent ligand ribbons by bridging ligand pairs of the adjacent ribbons (Figure 4; Figure 5a,b). CH⋅⋅⋅Br contacts (Table 1, Figure 5) again contribute to join ligand molecules.

**Figure 6:**
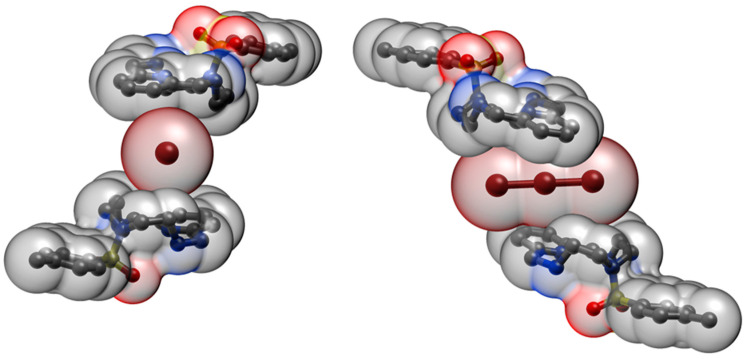
Space-fill views of Br_3_^−^ sandwiching between adjacent (H_2_L-Ts)^2+^ ligands.

The pyridine rings sandwiching this second Br_3_^−^ anion lie on parallel planes, but move relative to each other (Figure 5b) with an offset between the centroids of 2.66 Å. The two rings are antiparallel according to their polarity (Figure 2). The central Br1 atom of the sandwiched Br_3_^−^, lying on an inversion centre, is collinear with the centroids of the sandwiching pyridine rings and lies halfway from them, 3.84 Å from each (Figure 5). The closest distance between this Br atom and the pyridine planes is 3.60 Å, corresponding to an offset of 1.33 Å from the pyridine centroid, that is, Br1 is almost over the C2-C3 bond featuring a long η^2^-type interaction (C2⋅⋅⋅Br1 3.66 Å, C3-Br1 3.69 Å). The tribromide anion is not parallel to the pyridine rings (Figure 5b), and is arranged in such a way that two contiguous Br atoms are closer to the regions of positive electrostatic potentials of each sandwiching pyridine ring (Figure 2), while a third one keeps apart from the regions of negative electrostatic potential (the pyridine N atoms). Figure 6 shows two views of this tribromide anion sandwiched between the two pyridine groups. Although the position of this Br_3_^−^ anion is determined by the balance of a number of forces, the above features suggest that weak anion-π interactions with the pyridine rings might contribute to the overall stability of the crystal packing. Indeed, as we will see shortly, Hirshfeld surface analysis generated a typical fingerprint plot for these weak interactions, regardless of the interaction strength.

### 2.2. Detecting Anion-π Interaction from Fingerprint Plots

#### 2.2.1. Relevance and Method

Hirshfeld surface analysis provides a different perspective on crystal structures (cf. Materials and Methods section). While it is always instructive to look at the object of study under a different angle, it is also true that Hirshfeld surface analysis and fingerprint plots are especially useful in identifying relevant interaction types within a crystal structure or to quantify concepts like structural similarity. Both aspects were elucidated by Spackman and co-workers as they established this method of analysis [47,48,49]: we refer here to their demonstrative study showing periodicity in fingerprint plots for alkanes, hydrogen bonding in small molecules, polycyclic aromatic hydrocarbons (PAH) and halogenated derivatives [49]. Very marked tip-features in fingerprint plots have been shown to be typical of H-bonds, while, building on PAH studies, π-π interactions have been shown to also give recognisable fingerprint plot features [49].

Anion-π interaction, being a more niche topic, has not yet received much attention in this sense. Still, as anion-π interactions with N-heterocycles are among our interests, we have taken the chance to show here that a characteristic anion-π brand exists within fingerprint plots. We believe that establishing a bijective correspondence between an interaction type and its fingerprint plot image can be very important to aid deconvolution of fingerprint plot information. Moreover, as one of the strengths of Hirshfeld surfaces and fingerprint plots is being computationally inexpensive to access, future implementation of automated interaction recognition from fingerprint plots could be envisaged. The original proposers of the approach have already commented on the power of extracting information about interactions in a crystal lattice from fingerprint plots as a way to classify crystal structures on the basis of subtending interactions and easily provide evidence of structural differences and similarities [49].

The crystal structure of **1** is particularly convenient for this discussion, as it shows anion-π contacts both with benzene (tosyl) and pyridine rings. Yet a problem remains. How do we make sure that what we see in a single crystal structure is a general feature for anion-π interactions, given the extremely broad window of possibilities in terms of anion and N-heterocycle choices?

We circumvent the problem by re-analysing and presenting here evidence mainly from our previous studies. This allows us to refer to crystal structures clearly showing anion-π contacts which are fully described in classical terms in the original studies, so that full attention to the different perspective offered by fingerprint plots can be given here. However, to help the reader and make classic contact information easily available to her or him, we have included in the supplementary material a table (Table S1) which includes relevant interaction distances contributing to the H-bond tip (i.e., H-bond and CH⋅⋅⋅anion contacts) and anion-π region of fingerprint plots.

We will start the discussion from **1** featuring Br_3_^−^ interactions with a benzene (tosyl) and a pyridine ring. Our recent studies with iodides and Stoddart’s blue box [44,45,46], a pyridinium based cation, will help show that fingerprint plot features stay in place for pyridine if we move from Br_3_^−^ to I^−^, I_3_^−^ or I_5_^−^.

Our interest in anion complexes with pyrimidinic ligands will show that the same type of fingerprint plot features stays in place for the Br-based HgBr_4_^2−^ anion [50]. Maintaining the same ligand, HgCl_4_^2−^ behaves in the same way [50]. Replacing the halogen does not tamper with the observation, not even with the very different Co(CN)_6_^3−^ anion does it [51].

For *s*-triazines, the fitting example of a Br_3_^−^ complex from the literature will help confirm this observation [52]. The structure of the analogous BrIBr^−^ complex will further prove our point [52].

For *s*-tetrazines, which again are among our recent scientific interest, the structure of a Br^-^ complex will be used to verify this behaviour. Luckily enough, the same ligand provided crystals of anionic complexes for all halides (Br^−^, Cl^−^, FHF^−^ [39] and I^−^ [38]), which again can be used in this context.

In the end, the fact that one fingerprint plot feature is always present in the case of anion-π contacts and that it keeps constant when the aromatic ring (tosyl, pyridine, pyrimidine, *s*-triazine and *s*-tetrazine) and the anion (Br_3_^−^, Br^−^, I^−^, I_3_^−^, I_5_^−^, HgBr_4_^2−^, HgCl_4_^2−^, Co(CN)_6_^3−^, BrIBr^−^, Cl^−^, FHF^−^) are changed could be considered a good piece of evidence for a general behaviour.

#### 2.2.2. Analysis

**Figure 7:**
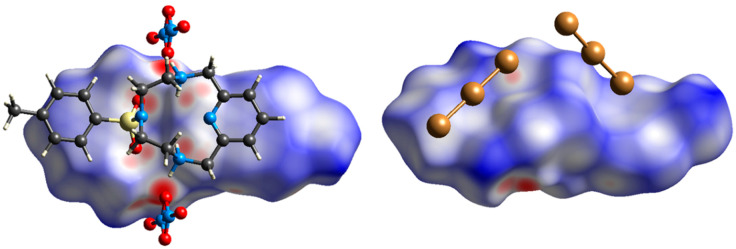
Depiction of (H_2_L-Ts)^2+^ Hirshfeld surface under a d_norm_ lens.

The (H_2_L-Ts)^2+^ Hirschfeld surface appearance under d_norm_ (≈ classical contact distance) lens is presented in Figure 7. As can be seen, the most prominent features are H-bond type interactions involving one side of the ligand. The other side, which appears to be interacting more mildly, is mainly in contact with Br_3_^−^ anions. 

**Figure 8:**
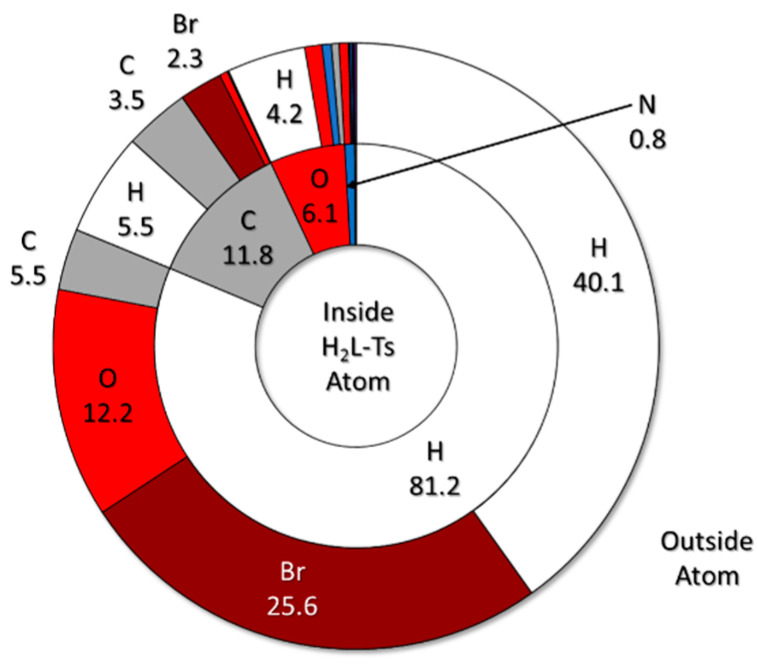
Overview of (H_2_L-Ts)^2+^ Hirshfeld surface percentage composition (complete data in tabular form are found in Table S2). Inner ring: internal composition of Hirshfeld surface, i.e., a depiction of (H_2_L-Ts)^2+^ molecular surface; outer ring: closest outer atom, i.e., atom in contact with (H_2_L-Ts)^2+^ Hirshfeld surface. Colour code: white: H; grey: C; blue: N; red: O; yellow: S; brown: Br.

A breakdown of (H_2_L-Ts)^2+^ Hirshfeld surface composition is presented in Figure 8. In percentage terms, most contacts involve the unavoidable H⋅⋅⋅H interactions (H is found at the periphery of most organic molecules) and the expected H⋅⋅⋅Br and H⋅⋅⋅O contacts indicative of both proper H-bonds and CH⋅⋅⋅Br/O contacts [49]. 

**Figure 9:**
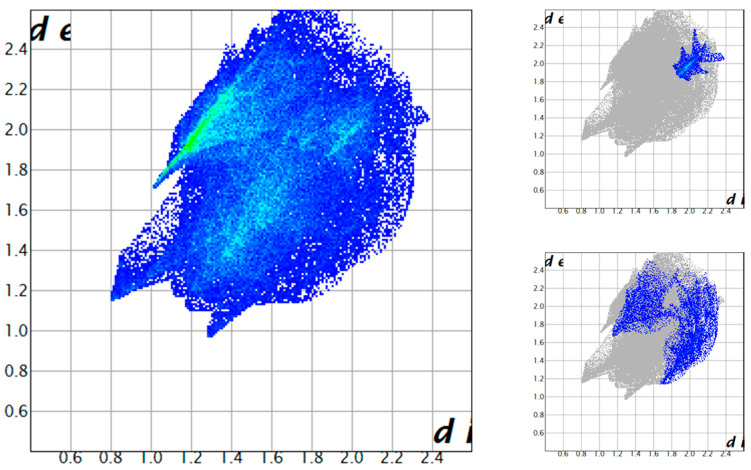
(H_2_L-Ts)^2+^ fingerprint plot. C-C contacts indicative of face to face π-π stacking and C-H wings are shown in top and bottom inset, respectively.

There are of course other contributions which have been previously identified and discussed [49]. These include H⋅⋅⋅C and reciprocal contacts, giving rise to the so called C-H wings in fingerprint plots and C⋅⋅⋅C interactions, implying the existence of face to face π-π stacking (Figure 3). The (H_2_L-Ts)^2+^ fingerprint plot and how these interactions reflect on it are illustrated in Figure 9. The relevance of stacking forces is well known, and nobody would generally challenge it. If the 3.5% of C⋅⋅⋅C contacts stands for a meaningful and well-recognised interaction, we see no reason for the 2.3% C⋅⋅⋅Br contacts not to be meaningful as well.

**Figure 10:**
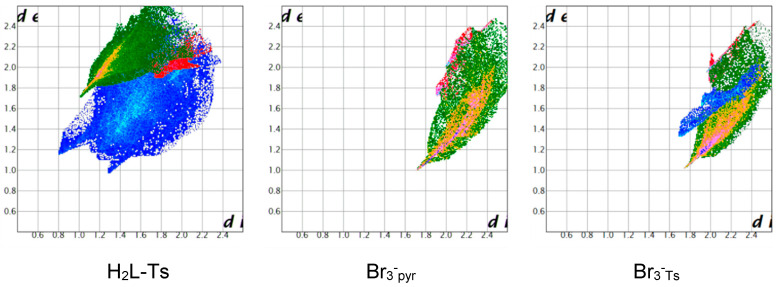
Ligand (H_2_L-Ts)^2+^ and Br_3_^−^ fingerprint plots. H-bond tip displayed in green hue. Anion-π contacts shown in red. Other interactions in standard fingerprint plot colouring. In the case of Br_3_^−^ interacting with tosyl group (Br_3_^−^_Ts_), the blue region is associated with the unusual Br_3_^−^⋅⋅⋅NO_3_^−^ contacts.

These contacts of course correspond to anion-π interactions. In Figure 10, we depict the (H_2_L-Ts)^2+^ fingerprint plot again. This time, H⋅⋅⋅Br contacts are highlighted in a green hue. The remainder of (H_2_L-Ts)^2+^ contacts with Br (C⋅⋅⋅Br and N⋅⋅⋅Br contacts) are shown in a red tone. As can be seen, the two regions corresponding to different types of interactions are separated and distinguishable, moreover, the same distinction can be made also on fingerprint plots of the anions. The anion-π region seems to be blurred and poorly defined, with differences among the two anions. Yet, as we will show, this is due to the simultaneous presence of two different Br_3_^−^ anion interacting with two different aromatic rings (the more the interactions of the same type, the more complicated their fingerprint plot image). 

**Figure 11:**
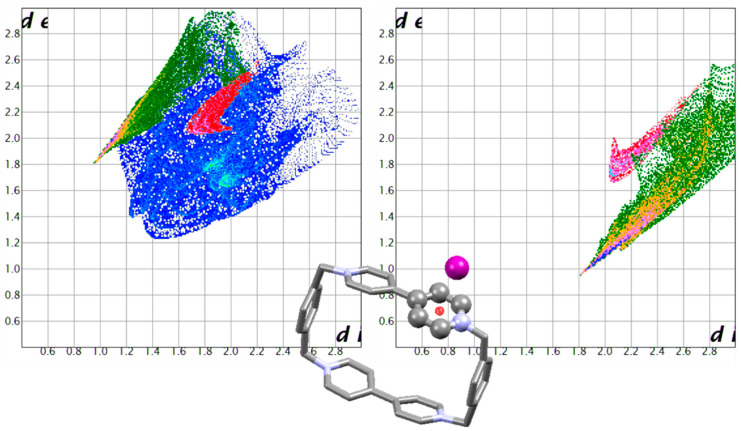
BB and I^−^ fingerprint plot in (ACN)_2_@(BB)I_4_ crystal structure (CSD refcode: HUDVOU [45]). Ligand and anion are interacting both via CH⋅⋅⋅I contacts (green) and anion-π interactions (red). The two contributions are markedly different and clearly recognisable. A brief view of the structure is presented for reference.

As examples of fingerprint plot features with pyridine ligands, we refer to our latest studies on the BB/I^−^/I_2_ system. As shown in Figure 11, for the most convenient (only ¼ of BB and a single I^-^ anion in the asymmetric unit) (ACN)_2_@(BB)I_4_ crystal structure [45], both the BB and I^−^ fingerprint plots show two markedly different regions belonging to I⋅⋅⋅H contacts and to anion-π interactions. These features remain unmodified, beyond the intrinsic specificity of each crystal structure and the complications involved in having more than one unique anion in the asymmetric unit, for BB/I^−^ crystal structures (5 structures [45]), BB/I_3_^−^ structure (1 structure [46]) and BB/I_5_^−^ structures (2 structures [44]) fingerprint plots are available in the original studies.

**Figure 12:**
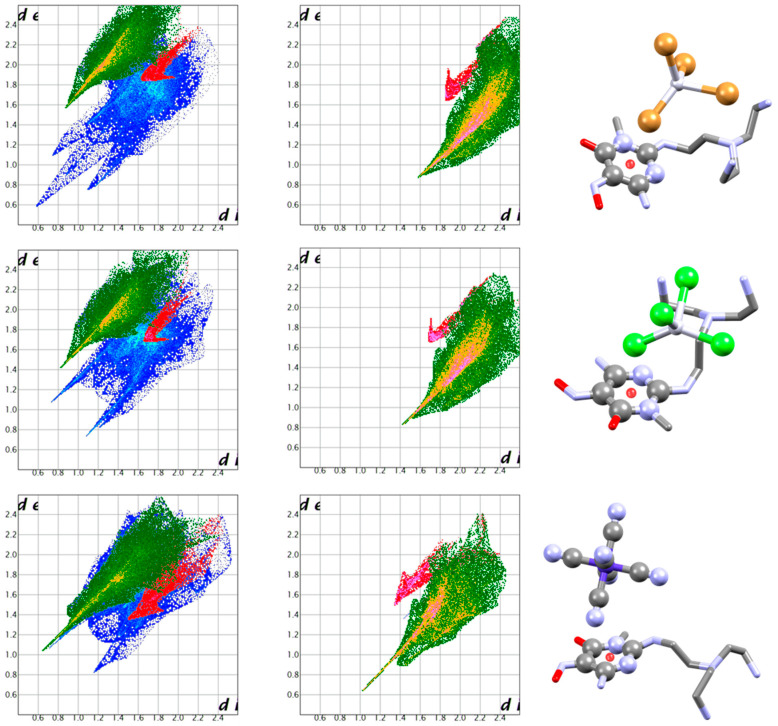
Complexes of the diprotonated 6-amino-2-((2-(bis(2-aminoethyl)amino)ethyl)amino)-3-methyl-5-nitrosopyrimidin-4(3*H*)-one ligand, bearing a functionalised pyrimidine residue, with HgBr_4_^2−^ (CSD refcode AVISEE [50]), HgCl_4_^2−^ (AVISII [50]) and Co(CN)_6_^3−^ (IDIKAJ [51]) anions. Ligand and anion fingerprint plots, together with a brief view of the structure, are presented for each one. All anions are bound via H-bonds (green) and anion-π interactions (red). The two contributions are markedly different and clearly recognisable.

For interactions with pyrimidine-based ligands, three well-documented systems showing anion-π contacts with an amino-nitroso pyrimidine are re-evaluated with Hirschfeld surface analysis [50,51]. The obtained fingerprint plots are reported in Figure 12. Again, H-bond type interactions and anion-π interactions give markedly recognised features. As the H-bond tip is modified from one structure to the other depending on the fine details of each individual structure, so does the fingerprint feature related to anion-π interactions. Yet, as we can distinguish a characteristic H-bond tip as a revealing feature, at the same time we must recognise the anion-π “swoosh” as equally telling. 

**Figure 13:**
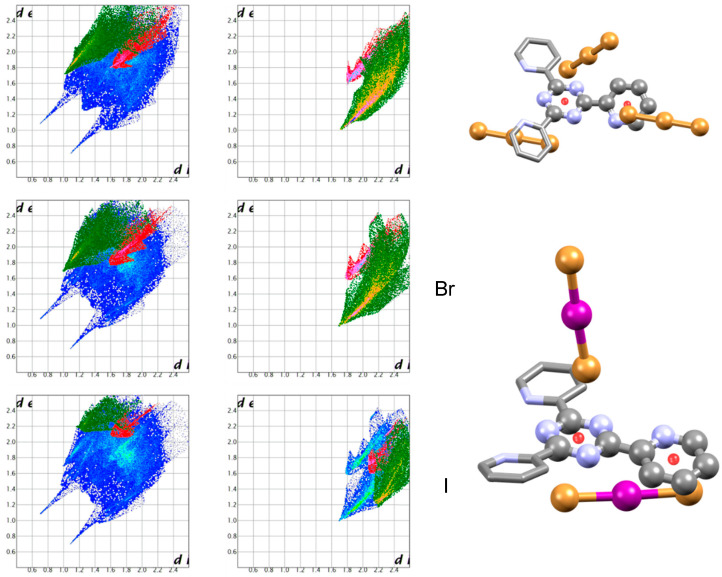
Complexes of the monoprotonated 2,4,6-tris(2-pyridyl)-1,3,5-triazine ligand with Br_3_^−^ (CSD refcode YOJDAD) and BrIBr^−^ anions (YOJDEH) anions [52]. Ligand and anion fingerprint plots, together with a brief view of the structure, are presented for each one. For YOJDEH, Br and I contribution to ligand and anion fingerprint plots have been highlighted separately. All anions are bound via H-bonds (green) and anion-π interactions (red). The two contributions are markedly different and clearly recognisable.

For interaction with *s*-triazines, a topic we did not address directly in our studies, we rely on two literature examples provided by Arca et al. [52] (Figure 13). These are of special interest as they share with the (H_2_L-Ts)(Br_3_)_1.5_(NO_3_)_0.5_ the unusual Br_3_^−^ anion (BrIBr^−^ is also closely related) and the fact that anion-π contacts involve 2 different N-heterocycles at once (in this case *s*-triazine and pyridine). Again, there is a recognisable anion-π region showing marked similarity with all the above examples. The YOJDEH crystal structure featuring the BrIBr^−^ anion helps show that polyatomic heteronuclear anions, in which different atoms are engaged in both anion-H and anion-π contacts, feature independently recognisable H-bond and anion-π fingerprint plot features. We remain able to distinguish each type of contacts despite the fact that Br and I are engaged in interactions of different strengths, as can be seen from the distinctive fingerprint plot regions developed for the two halides in Figure 13.

**Figure 14:**
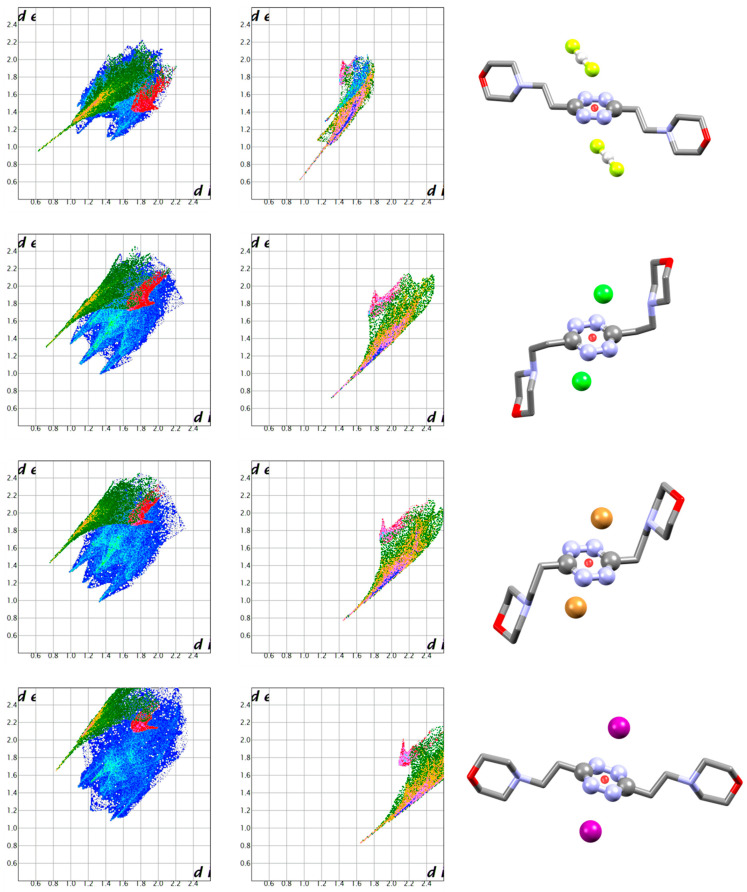
Complexes of the diprotonated 3,6-bis(2-morpholinoethyl)-1,2,4,5-tetrazine ligand with FHF^−^ (CSD refcode DETRIG), Cl^−^ (DETMOH), Br^−^ (DETMUN) and I^−^ (KAMLOC) anions [39]. Ligand and anion fingerprint plots, together with a brief view of the structure, are presented for each one. All anions are bound via H-bonds (green) and anion-π interactions (red). The two contributions are markedly different and clearly recognisable.

For *s*-tetrazine, maintaining a focus on Br-based anions for the sake of comparison, we present the interactions of the *s*-tetrazine ligand L2 with all halide anions (fluoride found as bifluoride anion, FHF^-^) (Figure 14) [38,39]. Again, anion-π contacts give rise to the characteristic and easily recognisable swoosh in both anion and ligand fingerprint plots, regardless of the interaction strength.

Identical fingerprint plot features connected with anion-π contacts for principal 6-memebered N heterocycles (pyridine, pyrimidine, s-triazine, s-tetrazine) and a good number of anions, Br-based (Br_3_^−^, Br^−^, HgBr_4_^2−^, BrIBr^−^) or not (I^−^, I_3_^−^, I_5_^−^, HgCl_4_^2−^, Co(CN)_6_^3^,Cl^−^, FHF^−^), demonstrate that an invariant anion-π signature exists in fingerprint plots, taking the shape of a famous logo. The reason behind recognisable fingertip plot features is very simple: each type of intermolecular interaction has its own topology. This is why H-bonds give sharp tips (there is a direction in which the electron density of the acceptor “pierces” the Hirshfeld surface of the donor) and how we can distinguish between CH⋅⋅⋅π and π⋅⋅⋅π contacts in PAH. In the very same manner, bringing an anion close to the centroid of an aromatic ring results in anion-C and anion-N contacts possessing an intrinsic pattern. The visualisation employed in this work, aimed at surpassing an element-by-element fingerprint plot deconvolution (the relative importance of anion-C and anion-N contacts remains dependent on the number of N atoms in the heterocycle), is effective at showing the subtending constant contribution. Anion-π fingerprint plot regions tend also to show finer details. The blurring observed for **1** arises from simultaneous Br contacts with different aromatic rings. The same can be said for Figure 13 and Co(CN)_6_^3−^ in Figure 12. All in all, recognising an interaction from a fingerprint plot feature is the first step towards its detailed analysis [49]. 

## 3. Materials and Methods 

### 3.1. General

The starting materials were high purity compounds purchased from commercial sources and were used as supplied. Ligand L (3,6,9,15-tetraazabicyclo[9.3.1]pentadeca-1(15),11,13-triene) was obtained as L⋅3HBr according to a reported procedure [5]. ^1^H NMR characterisation (Figure S1) of the sample used in this work showed the presence of about 2% of a monotosyslated ligand impurity, which remained after the detosylation procedure. The ^1^H NMR spectra were recorded using a Bruker Advance DPX300 (300 MHz, 7T) spectrometer (Bruker, Billerica, MA, USA), which operates at 300.13 MHz for ^1^H. Deuterium oxide (D_2_O) was used as solvent for the measurements, which were carried out at pD ca. 3.

### 3.2. Synthesis of (H_2_L-Ts)(Br_3_)_1.5_(NO_3_)_0.5_ (**1**)

Crystals of **1** were unexpectedly obtained in the attempt to prepare the nitrate salt of H_2_L^2+^ by treating with diluted HNO_3_ the above-cited sample of L⋅3HBr_,_ containing the small impurity (ca. 2%) of monotosylated ligand. 15 mg of L⋅3HBr were dissolved in 2 cm^3^ of 0.1 M HNO_3_ and the resulting solution was allowed to evaporate slowly at room temperature. The initial colourless solution turned pale orange during concentration to form a few crystals of (H_2_L-Ts)(Br_3_)_1.5_(NO_3_)_0.5_ with the same colour. Crystals were individually collected and air dried. The procedure was repeated four times until about 1 mg of compound was obtained for the elemental analysis. Elemental analysis: Anal. Calcd. for C_18_H_26_N_4.5_Br_4.5_O_3.5_S: C, 28.71%; H, 3.48%; N, 8.37%. Found: C, 28.62; H, 3.42; N, 8.31.

### 3.3. X-ray Structure Analyses

Orange crystals of **1** were used for X-ray diffraction analysis. A summary of the crystallographic data is reported in Table 2. The integrated intensities were corrected for Lorentz and polarisation effects and an empirical absorption correction was applied [53]. The structure was solved by SHELXS-97 [54]. Refinements were performed by means of full-matrix least-squares using SHELXL Version 2014/7 [54]. Data analysis suggested the presence of twinning. This possibility was verified, but the refined fraction for the twin component was extremely low (0.56%), and unable to significantly affect the final R value (0.1033 with twin law vs 0.1045 without twin law). As a consequence, we decided to neglect twinning and not to worsen the parameter/data ratio. The structure features positional disorder mainly affecting nitrate ions, and to a lesser extent, tribromides. The high content of electron-rich bromine atoms determined a significant series truncation errors, which were manifested by spurious maxima and minima in the electron density maps. All this is reflected in rather high values for anisotropic displacement parameters and agreement factors at the end of refinement. Nevertheless, the connectivity, checked by the Mogul Geometry Check module of Mercury (CSD), [55] is clear and the intermolecular interactions could be confidently analysed. CCDC 2010176 contains the crystallographic data for this structure.

### 3.4. Hirshfeld Surface Analysis

Hirshfeld surface can be defined as the region of space where the electron distribution of a theoretical collection of spherical atoms, representing the molecule under discussion (the promolecule), dominates the corresponding summation over the whole crystal depicted in the same manner (the procrystal). The mathematical definition, properties and usefulness of the Hirshfeld surface are found in dedicated literature [47,48]. The same treatment allows for the visualisation of a crystal structure through the lens of so-called fingerprint plots [49]. These are complete maps of external distance (d_e_) (i.e., the point-by-point distance of the nearest atom belonging to another molecule to the Hirshfeld surface of considered species) vs. internal distance (d_i_) (i.e., the point-by-point distance from a molecule’s Hirshfeld surface and the nearest atom belonging to the molecule itself) from the Hirshfeld surface under consideration, are colour-coded to show relative abundance (from blue: few contacts, to red: many contacts) of intermolecular contacts occupying d*i* × d*e* square bins of 0.01 × 0.01 Å^2^. Hirshfeld surface and fingerprint plots were calculated using the Crystalexplorer17 software [56]. The software allows one to highlight atom by atom contacts on the Hirshfeld surface and on fingerprint plots. This feature was exploited to further colour-code the presented figures so that the typical H-bond tip appears in a green hue and the anion-π contacts region (comprising anion-C and anion-N contact) is highlighted in a red hue. Since the anion-(C+N) contact region cannot be highlighted automatically by Crystalexplorer17, this was achieved manually by generating separate plots highlighting the different contact regions and overlaying them after altering their colours for displaying purposes. To preserve the original colour coding of fingerprint plots, which is valuable as it is informative about contact abundance, the colour change has been applied by using a colour shift filter on a graphic editor (namely, the freeware Inkscape [57]), so that all different shades, while shifted in tone, are maintained in the final picture.

## 4. Conclusions

The illustrative structure of (H_2_L-Ts)(Br_3_)_1.5_(NO_3_)_0.5_ has been elucidated, demonstrating once more the effectiveness of N-heterocycles in stabilising anionic complexes through anion-π interactions. While this is well-established and justified from an electrostatic viewpoint, the strength of these interactions is generally weaker the lower the number of N atoms in the ring. In this sense, observing the Br_3_^−^ anion-π interaction with pyridine, and even tosyl ring, is not commonplace. The anion itself is unusual, not to mention the bizarre Br_3_^−^⋅⋅⋅NO_3_^−^ interaction. 

On a general level, this structure prompted further examination of our previous studies and literature material concerned with anion-π interactions, which were reported before the popularisation of Hirshfeld surface analysis and fingerprint plot as tools. We demonstrated here, that, as for other well-known supramolecular forces (H-bonds, π-π stacking [49]), even anion-π contacts, beyond the specificity connected with individual crystal structures, generate an invariant and recognisable fingerprint plot feature. This remains true for different anions and different N-heterocycles and for strong and for weak interactions. The mark of the interaction takes the form of a characteristic swoosh, both in anion and ligand fingerprint plots. As devising a fingerprint plot profile for other supramolecular forces has immensely aided fingerprint plot analysis, we believe that appreciation of anion-π interactions at this level will be of high interest for the full recognition and further development of Hirshfeld surface-based tools.

## Figures and Tables

**Table 1:** Contacts in the crystal structure of (H_2_L-Ts)(Br_3_)_1.5_(NO_3_)_0.5_. Distances and angles involving hydrogen atoms in calculated positions are reported without e.s.d.

D⋅⋅⋅A	Distance (Å)	H⋅⋅⋅A	Distance (Å)	D-H⋅⋅⋅A	Angle (deg)
N3⋅⋅⋅O1	2.94(1)	H3B⋅⋅⋅O1	2.429	N3-H3B⋅⋅⋅O1	116.0
N1⋅⋅⋅O2	2.92(1)	H1A⋅⋅⋅O2	2.359	N1-H1A⋅⋅⋅O2	119.4
N3⋅⋅⋅O2′ (−x,−y,−z)	2.931(9)	H3B⋅⋅⋅O2′	2.317	N3-H3B⋅⋅⋅O2′	124.5
N1⋅⋅⋅O1′ (−x,−y,−z)	2.96(1)	H1A⋅⋅⋅O1′	2.389	N1-H1A⋅⋅⋅O1′	120.8
N1⋅⋅⋅O34	2.828(9)	H1B⋅⋅⋅O34	2.041	N1-H1B⋅⋅⋅O34	143.9
N3⋅⋅⋅O34′(−x,−y,−z)	2.83(1)	H3A⋅⋅⋅O34′	2.055	N3-H3A⋅⋅⋅O34′	142.4
N3⋅⋅⋅O32′(−x,−y,−z)	2.82(2)	H3A⋅⋅⋅O32′	2.140	N3-H3A⋅⋅⋅O32′	130.5
Br2′⋅⋅⋅C22 (−x + 1,−y + 1,−z)	3.90(1)	Br2′⋅⋅⋅H22	2.987	C22-H22⋅⋅⋅Br2′	162.2
Br2⋅⋅⋅C26 (−x,−y + 1,−z)	3.79(1)	Br2⋅⋅⋅H26	2.904	C26-H26⋅⋅⋅Br2	154.7
Br4⋅⋅⋅C17 (−x,−y + 1,−z)	3.73(1)	Br4⋅⋅⋅H17A	3.026	C17-H17A⋅⋅⋅Br4	128.8
Br4′⋅⋅⋅C19 (−x + 1,−y + 1,−z)	3.71(1)	Br4′⋅⋅⋅H19B	2.986	C19-H19B⋅⋅⋅Br4′	131.3
Br5⋅⋅⋅C16	3.89(1)	Br5⋅⋅⋅H16B	3.004	C16-H16B⋅⋅⋅Br5	150.2
Br5⋅⋅⋅C13	3.680(7)	Br5⋅⋅⋅H13A	2.812	C13-H13A⋅⋅⋅Br5	146.7
Br5⋅⋅⋅C25 (−x,−y + 1,−z)	3.89(2)	Br5⋅⋅⋅H25	2.947	C25-H25⋅⋅⋅Br5	173.3

**Table 2:** Crystal data and refinement parameters for (H_2_L-Ts)(Br_3_)_1.5_(NO_3_)_0.5_ (**1**).

Empirical Formula	C_18_H_26_Br_4.5_N_4.5_O_3.5_S
Formula weight	753.09
Temperature (K)	100
Crystal system	triclinic
Space group	P-1
	
a (Å)	9.8426(9)
b (Å)	11.428(1)
c (Å)	13.413(1)
α (°)	105.072(3)
β (°)	99.065(3)
γ (°)	115.435(3)
Volume (Å^3^)	1251.3(2)
Z	2
Independent reflections/R(int)	4423/0.0625
μ (mm^−1^)	9.838 (Cu-Kα)
R indices [I > 2σ(I)] ^a^	R1 = 0.1045
	wR2 = 0.2905
R indices (all data) ^a^	R1 = 0.1083
	wR2 = 0.2943

^a^ R1 = Σ || Fo| − |Fc|| / Σ |Fo|; wR2 = [ Σ w(Fo^2^ − Fc^2^)^2^ / Σ wF.0

## Supplementary Materials

The following are available online at https://www.mdpi.com/1420-3049/25/14/3155/s1, Table S1: Breakdown of main anion-ligand interactions contributing to H-bond tip and anion-π swoosh as found in crystal structures presented in Figure 11, Figure 12, Figure 13 and Figure 14. Full information can be retrieved from original publications and/or directly from CSD database, Table S2: Breakdown of (H_2_L-Ts)^2+^ Hirshfeld surface in (H_2_L-Ts)(Br_3_)_1.5_(NO_3_)_0.5_ crystal structure, Figure S1: 1H NMR spectrum recorded on a D_2_O solution (pD ca. 3) of L⋅3HBr. a) Aliphatic and aromatic signals; b) an enlarged detail of aromatic signals. The spectrum allows to detect and quantify the presence of an impurity of L-Ts (monotosylated ligand) in about 2%. Crystallographic information file is available from the CCDC, reference number 2010176.
