# Supplementary material for: Stabilisation of Exotic Tribromide (Br3−) Anions via Supramolecular Interaction with a Tosylated Macrocyclic Pyridinophane. A Serendipitous Case
Source: Molecules. 2020 Jul 10;25(14):3155. doi: 10.3390/molecules25143155 (PMC7396983; doi:10.3390/molecules25143155)
Supplement: Supplementary file 1 [file molecules-25-03155-s001.pdf]

Supplementary Materials

Stabilisation of Exotic Tribromide (Br_3^-) Anions via Supramolecular Interaction with A Tosylated Macrocyclic Pyridinophane. A Serendipitous Case.

Álvaro Martínez-Camarena [1,†], Matteo Savastano [2,†], Carla Bazzicalupi [2], Antonio Bianchi [2,*] and Enrique García-España [1,*]

[1] ICMol, Department of Inorganic Chemistry, University of Valencia, C/Catedrático José Beltrán 2, 46980, Paterna, Spain.; alvaro.martinez@uv.es
[2] Department of Chemistry "Ugo Schiff", University of Florence, Via della Lastruccia, 3-13, 50019 Sesto Fiorentino, Italy; matteo.savastano@unifi.it (M. S.); carla.bazzicalupi@unifi.it (C. B.).
* Correspondence: antonio.bianchi@unifi.it (A.B.); Enrique.Garcia-Es@uv.es (E. G-E.).
† These authors contributed equally.

Academic Editor: György Szöllösi

Received: 18 June 2020; Accepted: 08 July 2020; Published: date

Table of anion-ligand interactions for the crystal structures analysed in this work. Table of Hirshfeld surface data for the $(H_2L\text{-}Ts)(Br_3)_{1.5}(NO_3)_{0.5}$ crystal structure. ^1H NMR spectrum of the ligand L showing the presence of ca. 2% impurity of L-Ts.

Table S1. Breakdown of main anion-ligand interactions contributing to H-bond tip and anion-π swoosh as found in crystal structures presented in Figures 11-14. Full information can be retrieved from original publications and/or directly from CSD database.

**Crystal Structure** (Figure)	**Anion**[a]	**H-bond tip** (contact distance range)†[b]	**Anion-π swoosh** (contact distance range)†[c]
**HUDVOU** (Fig. 11)	I^- (1)	5 CH···I contacts (3.82-4.03)	1 anion-π contact (3.61)
**AVISEE** (Fig 12)	$HgBr_4^{2-}$ (1)	3 CH···Br contacts (3.80-3.94)<br>8 NH···Br contacts (3.38-3.63)	1 anion-π contact (3.35)
**AVISII** (Fig. 12)	$HgCl_4^{2-}$ (1)	3 CH···Cl contacts (3.45-3.57)<br>6 NH···Cl contacts (3.23-3.49)	1 anion-π contact (3.13)
**IDIJAJ** (Fig. 12)	$[Co(CN)_6]^{3-}$ (2)[d]	6 NH···N contacts (2.81-3.03)<br>6 OH···N contacts (2.68-2.84)	2 anion-π contacts (2.78-3.44)
**YOJDAD** (Fig. 13)	Br_3^- (1)	4 CH···Br contacts (3.60-3.88)	3 anion-π contacts (3.25-3.83)
**YOJDEH** (Fig. 13)	$BrIBr^-$ (1)	4 CH···Br contacts (3.49-3.84)[e]	2 anion-π contacts (3.28-3.58)[e]
**DETRIG** (Fig. 14)	F^-/FHF^- (1 each)	10 CH···F contacts (3.25-3.54)<br>3 O_WH···F contacts (2.36-2.57)	1 anion-π contact (3.01)
**DETMOH** (Fig. 14)	Cl^- (1)	3 CH···Cl contacts (3.52-3.71)<br>1 NH···Cl contact (3.06)	1 anion-π contact (3.31)
**DETMUN** (Fig. 14)	Br^- (1)	5 CH···Br contacts (3.60-3.99)<br>1 NH···Br contacts (3.23)	1 anion-π contact (3.41)
**KAMLOC** (Fig. 14)	I^- (2)[d]	5 CH···I contacts (4.01-4.22)<br>2 NH···I contacts (3.45-3.48)	2 anion-π contacts (3.67-3.70)

† All distances in Å; [a] number of non-equivalent anions within the crystal structure in brackets; [b] given distance range is intended as anion-heavy (non-hydrogen) atom distance; [c] all distances given as anion-centroid distances; [d] in these cases, qualitative image shown in the text represents the closest anion-π contact within the crystal structure; [e] contacts with I cannot be classified as short, yet CH···I and I-π interactions are till distinguishable in the fingerprint plot.

Table S2. Breakdown of $(H_2L\text{-}Ts)^{2+}$ Hirshfeld surface in $(H_2L\text{-}Ts)(Br_3)_{1.5}(NO_3)_{0.5}$ crystal structure

$(H_2L\text{-}Ts)^{2+}$							
**Inside Atom**	**Outside Atom**						**Total**
	**Br**	**S**	**N**	**H**	**O**	**C**	
**C**	2.3	.	0.1	5.5	0.4	3.5	11.8
**H**	25.6	.	.	40.1	12.2	3.3	81.2
**N**	0.1	0.1	.	.	0.5	0.1	0.8
**O**	0.0	.	0.5	4.2	0.9	0.4	6.1
**S**	.	.	0.1	.	.	.	0.1
**Total**	28.1	0.1	0.7	49.8	14.1	7.3	

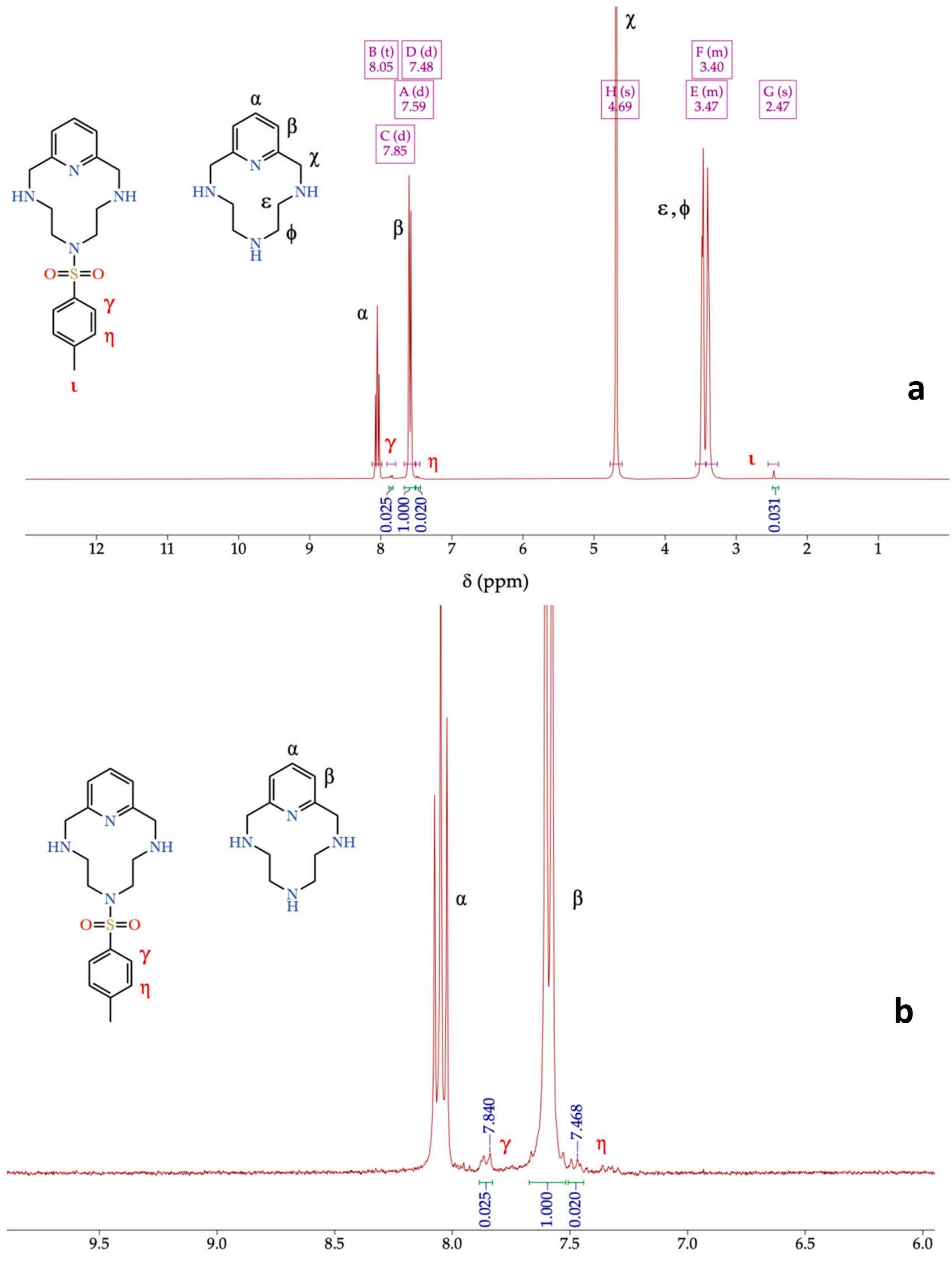

Figure S1. ^{1}H NMR spectrum recorded on a D_2O solution (pD ca. 3) of L·3HBr. a) Aliphatic and aromatic signals; b) an enlarged detail of aromatic signals. The spectrum allows to detect and quantify the presence of an impurity of L-Ts (monotosylated ligand) in about 2%.
